# Pediatric pulmonary hemorrhage observed in non-vascular and vascular Ehlers–Danlos syndrome

**DOI:** 10.1186/s13023-025-03858-2

**Published:** 2025-07-01

**Authors:** Rong Yang, Haiming Yang, Chen Shen, Xingfeng Yao, Jinrong Liu, Huimin Li, Shunying Zhao

**Affiliations:** 1 Department of Respiratory Medicine, Beijing Children’s Hospital, Capital Medical University, National Center for Children’s Health, No.56 Nanlishi Road, Beijing, 100045 China; 2 Laboratory of Respiratory Diseases, Beijing Key Laboratory of Pediatric Respiratory Infection Diseases, Key Laboratory of Major Diseases in Children, Ministry of Education, National Clinical Research Center for Respiratory Diseases, National Center for Children’s Health, Beijing Pediatric Research Institute, Beijing Children’ s Hospital, Capital Medical University, Beijing, China; 3 Department of Pathology, Beijing Children’s Hospital, Capital Medical University, National Center for Children’s Health, No.56 Nanlishi Road, Beijing, China

**Keywords:** Children, Ehlers–Danlos syndrome, Pulmonary hemorrhage

## Abstract

**Background:**

Ehlers–Danlos syndrome (EDS) is a heterogeneous group of heritable connective tissue disorders with varying features depending on the EDS subtype. EDS is associated with various respiratory manifestations. Pulmonary hemorrhage has been previously reported in vascular EDS (vEDS); however, this manifestation remains not particularly well-defined in other subtypes of EDS. This study extends the clinical understanding of EDS, particularly non-vascular EDS, and expands etiological spectrum of pulmonary hemorrhage in children.

**Methods:**

We retrospectively analyzed the records of patients diagnosed with EDS between January 2020 and November 2024 at our institute. All clinical data was extracted from the electronic medical records, including clinical presentation, physical examination, family history, and chest computed tomography scans. EDS was confirmed based on clinical manifestations, pathological biopsies, immunohistochemistry, immunofluorescence staining, and genetic testing.

**Results:**

Our study identified eight patients with EDS who presented with pulmonary hemorrhage. Among these eight patients, nine gene mutations were identified, including four in *COL3A1*, two in *COL1A1*, one in *COL1A2*, one in *TNXB*, and one in *COL4A2*. We identified the mutations: IVS44 + 1G→A and c.1550 C > T (p. Pro517Leu) of *COL3A1* gene as two novel mutations associated with vEDS. And we added pathogenic evidences of the mutations c.1550 C > T (p. Pro517Leu) and c.3133G > A (p. Ala1045Thr) in *COL3A1* gene.

**Conclusions:**

Two novel and two pathogenic mutations in *COL3A1* gene associated with vEDS, *COL1A1*, *COL1A2*, *TNXB* gene mutations of non-vascular types underlying EDS and *COL4A2* gene associated with collagen synthesis were found in patients presenting with pulmonary hemorrhage. These findings would enhance clinical recognition of EDS and provide a sound basis to recommend that children with pulmonary hemorrhage be routinely examined for joint and skin hyperextension.

**Supplementary Information:**

The online version contains supplementary material available at 10.1186/s13023-025-03858-2.

## Background

Ehlers–Danlos syndrome (EDS) is a heterogeneous group of heritable connective tissue disorders characterized by skin hyperextensibility, joint hypermobility, and tissue fragility. To date, a total of 14 EDS subtypes and 20 causative genes have been identified [1, 2], which are primarily involved in collagen and extracellular matrix synthesis/maintenance. Depending on the EDS subtype, respiratory manifestations may include dyspnea, dysphonia, asthma, sleep apnea, and reduced respiratory muscle function [3]. The most frequent bleeding symptoms of EDS include abrasions, muscle hematomas, menorrhagia, nose bleeding, oral bleeding, and bleeding after tooth extraction [4]. Patients with vascular EDS (vEDS) have an increased risk of pneumothorax, hemothorax, intrapulmonary bleeding, cysts, and non-malignant fibrous nodules [5]. However, pulmonary hemorrhage in vEDS remains poorly reported; diffuse alveolar hemorrhage (DAH) has only been reported in five cases [6–10], hematoma in five cases [11–15], and focal hemorrhaging in three cases [16–18]. In other subtypes of EDS, pulmonary hemorrhage has hardly been reported. Herein, we identified two novel mutations in *COL3A1* gene associated with vEDS, and added pathogenic evidences of two mutations in *COL3A1* gene, four gene mutations of non-vascular types underlying EDS in four patients, and one gene mutation associated with collagen synthesis in one patient, all in patients presenting with pulmonary hemorrhage. This study extends the clinical understanding of EDS, particularly non-vascular EDS, and expands etiological spectrum of pulmonary hemorrhage in children.

## Methods

### Patient cohort

This study involved a retrospective analysis of the records of patients diagnosed with EDS between January 2020 and November 2024. Differential diagnosis was performed in each patient, excluded diagnosis such as vasculitis, connective tissue disease, and autoinflammatory diseases. Detailed information on the laboratory tests used for differential diagnosis is presented in Additional File 1: Supplementary Table (1) Clinical data of each patient, including clinical manifestations, physical examination findings, family history, chest computed tomography (CT) scans, and pathological findings, were extracted from electronic medical records. Details of each patient subjected examinations and experiments were shown by Additional File 1: Supplementary Table (2) This study was approved by the Ethics Committee of Beijing Children’s Hospital, Capital Medical University (Ethical approval number: [2022]-E-010-Y).

### Immunohistochemistry staining

Immunohistochemistry (IHC) was used to quantify the expression of proteins of interest in the patient’s lung and skin tissue. Control tissues were obtained by puncture from non-EDS patients. Formalin-fixed and paraffin-embedded skin tissue sections with a thickness of 5 μm were obtained from patients with EDS (*n* = 3, patient 1, 2, and 7) and controls (*n* = 2). Tissue samples were stained separately for *COL3A1* and *TNXB* using polyclonal rabbit anti-COL3A1 (NB600-594SS, Bio-Techne, Minneapolis, Minn, USA) and anti-TNXB (PB9833-10ug, Boster, San Jose, CA, USA) antibodies. A polyclonal Goat Anti-Rabbit secondary antibody (ab6721, Abcam, Cambridge, UK) was used to detect the target primary antibodies. Counterstaining was performed using hematoxylin, and the slides were scanned using a digital scanner. All tissue sections were stained together to avoid batch effects. All samples were coded before the experiments, and identifying information was blinded to the investigators.

### Immunofluorescence analysis of *COL1A1* expression in human dermal fibroblasts

Skin fibroblasts from patient 4 and a child with normal-looking skin (kindly provided by the Department of Neurology) were primarily cultured from tissue blocks following skin puncture. Informed consent was obtained from the patients or their guardians. Fibroblasts were propagated in Dulbecco’s modified Eagle medium supplemented with 10% FBS and 1% antibiotics in a cell culture incubator at 37 °C with 5% CO_2_. The growth medium was refreshed every 2 days, and sub-culturing was conducted at 80–90% confluence until fibroblasts had been passaged 5–7 times.

Fibroblasts were fixed in 4% paraformaldehyde, washed three times with PBS, and permeabilized for 20 min at 25 °C in 0.5% Triton X-100. Next, cell samples were incubated with primary antibodies (rabbit anti-COL1A1, 72026T, CST, Boston, Mass, USA) for 40 min at 37 °C. Alexa Fluor 488 anti-rabbit antibody was used as the secondary antibody. Finally, the stained DNA was used to obtain confocal and high-resolution images.

### Genetic sequencing and variant assessment

Genomic DNA samples were prepared for each patient, and exonic DNA sequences were enriched. Illumina and base calling methods were used for sequencing and raw data evaluation. The following three categories of data sequences were excluded from the analysis: those with quality scores below 20, those exhibiting synonymous variations, and single-nucleotide polymorphisms with a minor allele frequency exceeding 5%. The pathogenicity of missense mutations was assessed using SIFT, PolyPhen-2, and Mutation Taster. Meanwhile, variants were evaluated in accordance with the guidelines of the American College of Medical Genetics and Genomics (ACMG).

## Results

### Demographic and clinical features of patients

Patients' demographics and clinical features are presented in Table 1. Diffuse hemorrhagic manifestations were noted in six cases, localized hematomas in two, and hemoptysis in four. Cough occurred in three patients, and wheezing occurred in two. Pallors were noted in three patients, fatigue in four, poor activity tolerance in one, and chest pain in one. Hemosiderin cells were observed in alveolar lavage and gastric fluid specimens from all patients. All patients exhibited complications, albeit with various severities. Specifically, patient 1 exhibited pneumothorax, pleural effusion, gastrointestinal bleeding, diarrhea, tricuspid regurgitation, and bone dysplasia. Both patients 2 and 3 showd similar facial features. Patients 4, 5, and 6 experienced cardiac complications, including arrhythmias and atrial septal defects. Patient 7 had a history of hip dislocation. Patients 5 and 8 presented with growth retardation. Patient 8 had a history of pneumothorax, pleural effusion, pericardial effusion, and skeletal developmental abnormalities.

**Table 1:** Demographic and clinical characteristics of the enrolled patients

Patient No.	Age at diagnosis, y	Sex	Symptom								Clinical		examination		Family history	Imaging findings						
			Cough	Phlegm	Wheezing	Hemoptysis	Pallor	Fatigue	Poor activity endurance	Chest pain	Clubbing digits	Joints hypermobility	Joints swelling	Skin hyperextensibility		Ground glass opacities	Parenchymal lesions	Pneumothorax	Cavity formation	Cysts	Pulmonary vascular abnormalities	Mediastinal lymph node enlargement
1	13.75	F				+				+		+		+		+	+	+	+			
2	7.75	F				+	+	+				+		+	+	+	+					
3	2.83	F			+		+	+				+		+	+	+	+					
4	5.55	M	+	+		+						+		+	+	+	+		+			+
5	12.00	F	+	+					+		+	+		+	+	+				+	+	+
6	6.83	F					+	+				+		+	+	+						
7	13.68	M				+						+		+	+	+	+		+	+	+	
8	3.83	F	+		+			+			+	+	+	+		+	+					

### Imaging findings

**Fig. 1:**
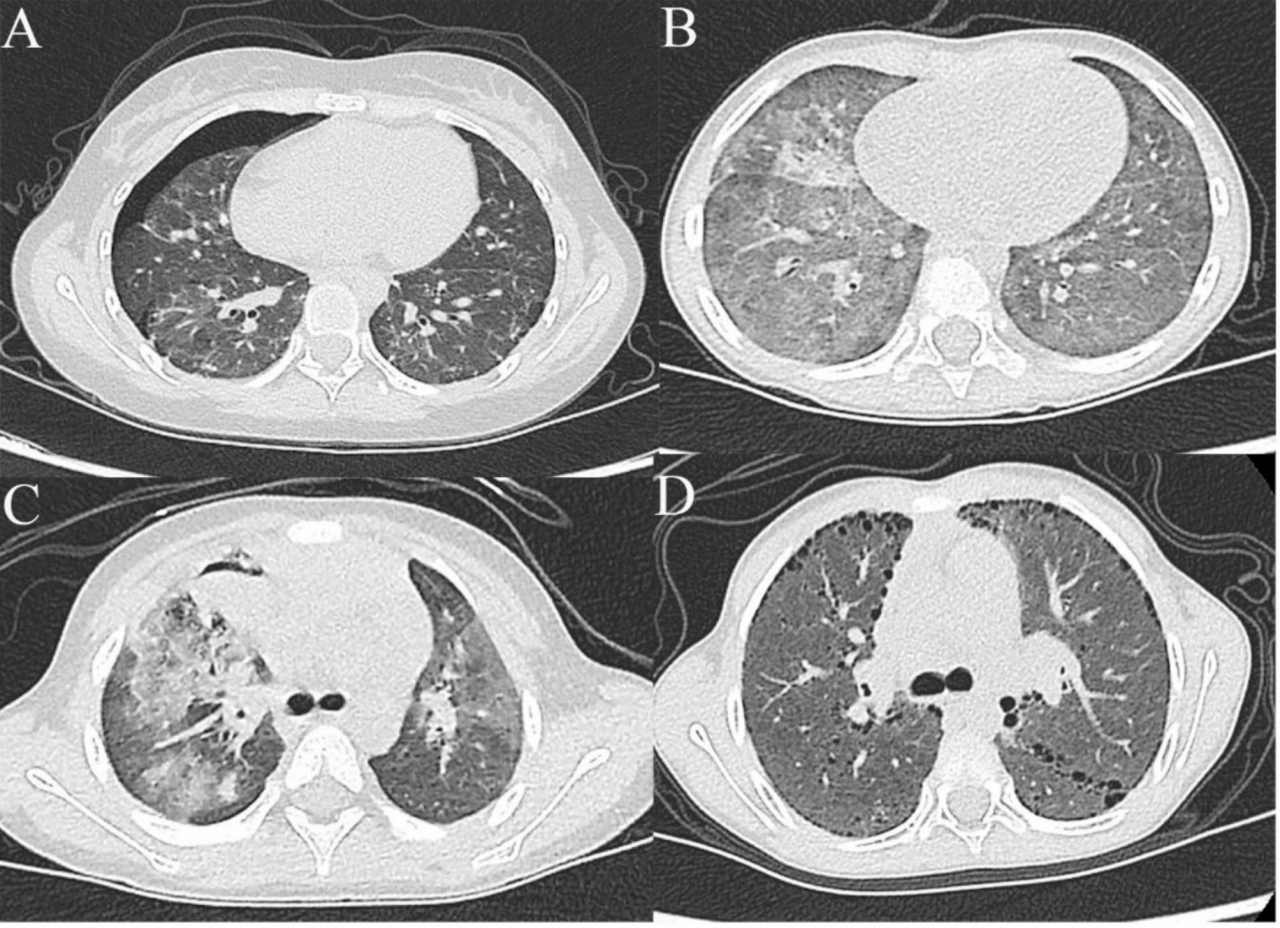
Imaging findings in patients 1, 3, 4, and 5, including: (**A**) pneumothorax and ground-glass opacities (GGOs) in patient 1, (**B**) multifocal GGOs in patient 3, (**C**) cavity formation, parenchymal lesions, and GGOs in patient 4, (**D**) small nodular GGOs and cysts in patient 5

Chest CT scans were performed on all eight patients for evaluation. All scans revealed abnormalities (Fig. 1); one patient had unilateral lung involvement, whereas the others had bilateral involvement. Six patients had multifocal ground-glass opacities (GGOs), and two patients had small nodular ground-glass opacities. One patient had pneumothorax, two patients had cavity formation within limited parenchymal lesions, and two patients had cysts. One patient had lobular septal thickening, one had calcified nodules, two had pulmonary vascular abnormalities, and two had mediastinal lymph node enlargement.

### Pathology results

**Fig. 2:**
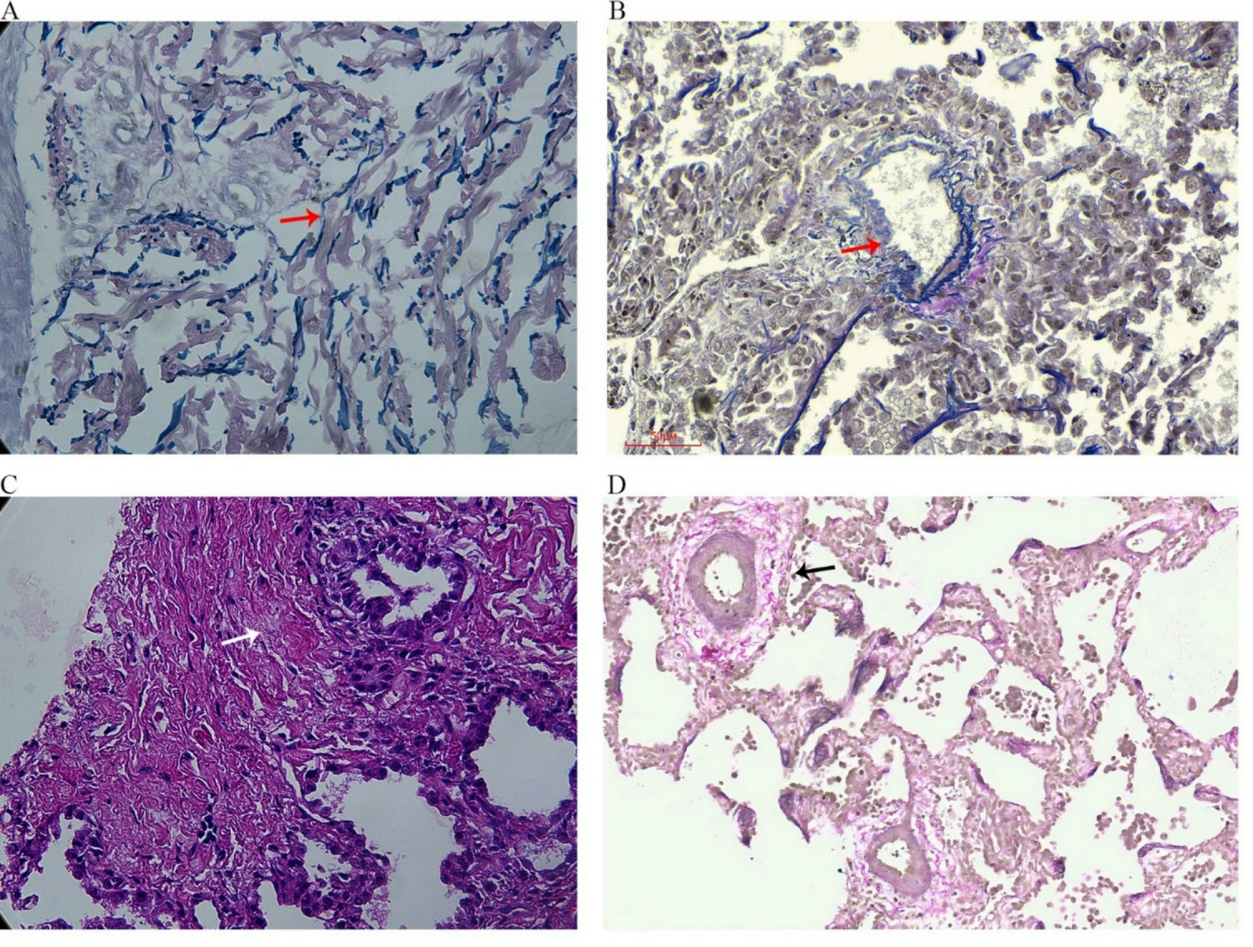
Pathological results in patients 2, 4, 7, and 8, showing (**A**) broken elastic fibers of the dermis in patient 2, (**B**) broken elastic fibers of the vascular wall in patient 4, (**C**) fibrous nodules in the left lung in patient 7, (**D**) broken elastic fibers of the vascular wall and uneven thickening of the interstitial great blood vessel walls in patient 8. ((**A**), (**B**), (**D**) Elastic van Gieson stain ×40;(**C**) hematoxylin-eosin stain ×40)

In this study, the dermatopathological findings of four cases revealed loose and proliferating collagen fibers in the dermis, whereas three cases showed focal breaks in the elastic fibers after staining. Lung pathology revealed a widening of the alveolar septa, a small amount of lymphoid infiltration, and uneven thickening or local destruction of the great interstitial blood vessel walls in all patients. All cases showed hemosiderin cells that were visible in the alveolar lumen. The findings are illustrated in Fig. 2.

### IHC

**Fig. 3:**
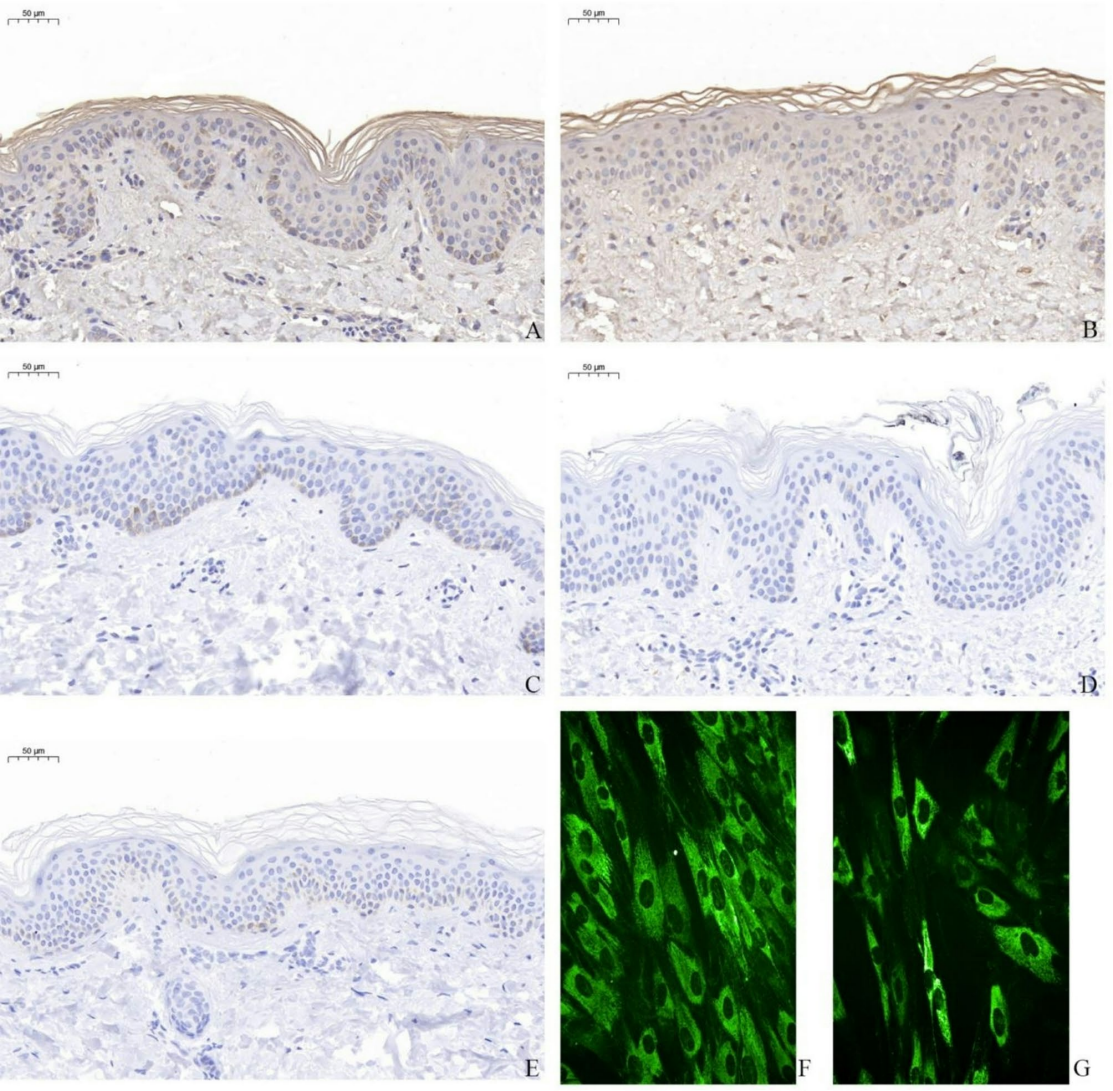
IHC results in patients 1, 2, 7 and Immunofluorescence staining Patient 4. Immunohistochemistry results in patient 7 (**B**) showed collagen expression was widely reduced compared with controls (**A**) in basement membrane. The expression of type III collagen from patients 1(**D**) and 2(**E**) significantly lower compared with controls (**C**). There are large quantities of type I collagen, uniformly distributed in normal fibroblasts (**F**). In contrast, *COL1A1* expression was reduced and significantly unevenly distributed in the fibroblasts of Patient 4(G). ((**A**) and (**B**), *TNXB* antibody IHC stain ×40; (**C**), (**D**) and (**E**), *COL3A1* antibody IHC stain ×40; (**F**) and (**G**), immunofluorescence stain ×20)

IHC staining showed significantly lower expression of type 3 collagen in the skin samples of patients 1 and 2 than in the control samples (Fig. 3C, D, and E). Dermatopathological IHC results from patient 7 showed that collagen expression in the basement membrane was significantly reduced compared with the controls, affecting tenascin XB (Fig. 3A and B).

### Immunofluorescence analysis

Immunofluorescence staining demonstrated large quantities of type I collagen uniformly distributed in normal fibroblasts (Fig. 3F). In contrast, type I collagen was reduced and significantly unevenly distributed in the fibroblasts of patient 4 (Fig. 3G).

### Genetic testing findings

All gene mutations in the eight patients are shown in Table 2. Pathogenicity analysis of each mutation is detailed in the Discussion section.

**Table 2:** Genetic testing results in the enrolled patients

Patient no.	Subtype	Gene	Complementary DNA change (protein change)	dbSNP	Source	MAF	ACMG		Location in the peptide chain	Forms affecting function
1	vEDS	COL3A1	c.3255 + 1(IVS44)G > A	RS587779480	De novo	None	Path	PVS1 + PS2 + PM2	C-terminal prepeptide initiation	Exon 44 jumps or intron retention
2	vEDS	COL3A1	c.1804C > A(p. P602T)	RS35795890	M	0. 0051	LP	PM1 + PM5 + PP3 + PP4	Within the Triple Helix region	Hydroxyproline production is blocked, disrupting the hydrogen bonding network
		COL3A1	c.1550C > T(p. P517L)	RS142085247	M	0. 001	LP	PM1 + PM2 + PP1 + PP3 + PP4	Within the Triple Helix region	Hydroxyproline production is blocked, disrupting the hydrogen bonding network
3	vEDS	COL3A1	c.3133G > A(p.A1045T)	Rs149722210	P	0. 0053	VUS	PM2 + PP3 + PP4	The tail of the triple helix region (adjacent to the C-terminal pre-peptide)	Interference with prepeptide folding, block interchain disulfide bond formation
4	aEDS	COL1A1	c.2573C > G(p.Ala858Gly)	RS550053089	M	0. 0001	VUS	PM2 + PP3 + PP4	Within the Triple Helix region	Increase directional heterogeneity in collagen bending
5	aEDS	COL1A1	c.3979G > A(p.Gly1327Ser)	RS147104425	P	None	VUS	PM2 + PP4	Fibrillar collagen NC1 structural domain in the C-terminal prepeptide region	Interference with prepeptide folding, block interchain disulfide bond formation
6	aEDS	COL1A2	c.3313G > A(p.G1105S)	RS139851311	P	0. 0015	LP	PM1 + PM2 + PP3 + PP4	Located at the end of the triple helix region	Interference with prepeptide folding, block interchain disulfide bond formation
7	clEDS	TNXB	c.211G > T(p. V71L)	Rs201922477	M	None	LP	PM1 + PM2 + PP3 + PP4	N-terminal oligomerized structural domain	Regulate collagen deposition by fibroblasts
8	vEDS	COL4A2	c.4691C > A(p. S1564Y)	NA	P	0.0000083	LP	PM1 + PM2 + PP3 + PP4	C-terminal structure	Affect the proper assembly and maturation of collagen fibers

vEDS = vascular Ehlers-Danlos syndrome; aEDS = Arthrochalasia EDS; clEDS = Classical-like EDS; ACMG = American College of Medical Genetics and Genomics; dbSNP = Single Nucleotide Polymorphism Database; NA = not applicable; M = maternal; *P* = paternal; Path = pathogenic; LP = likely pathogenic; VUS = variant of uncertain significance

### Treatment

Each patient was asked to restrict their activities, and pulmonary hemorrhage significantly improved in patients 1 and 6 after activity restriction. Patient 4 was effectively treated with bronchial artery embolization (BAE), while glucocorticoids were administered to all patients in the acute phase until a definitive diagnosis was obtained. In addition, patients were advised to take appropriate vitamin C and E supplements.

## Discussion

Our study described eight patients with pulmonary hemorrhage caused by vEDS and non-vascular EDS. A total of nine gene mutations were identified, including four mutations in the *COL3A1* gene, two in the *COL1A1* gene, one in the *COL1A2* gene, one in the *TNXB* gene, and one in the *COL4A2* gene. The *COL4A2* gene mutation affects vascular endothelium integrity, causing pulmonary hemorrhage.

Type III collagen is a homotrimer of three identical alpha chains encoded by *COL3A1* associated with vEDS. Forty-four exons encode the triple-helical domain of *COL3A1* [19]. Patient 1’s diagnosis of vEDS was definite based on the clinical signs and dermatopathological findings. This patient carried the mutation IVS44 + 1G→A, which is pathogenic according to ACMG.

**Fig. 4:**
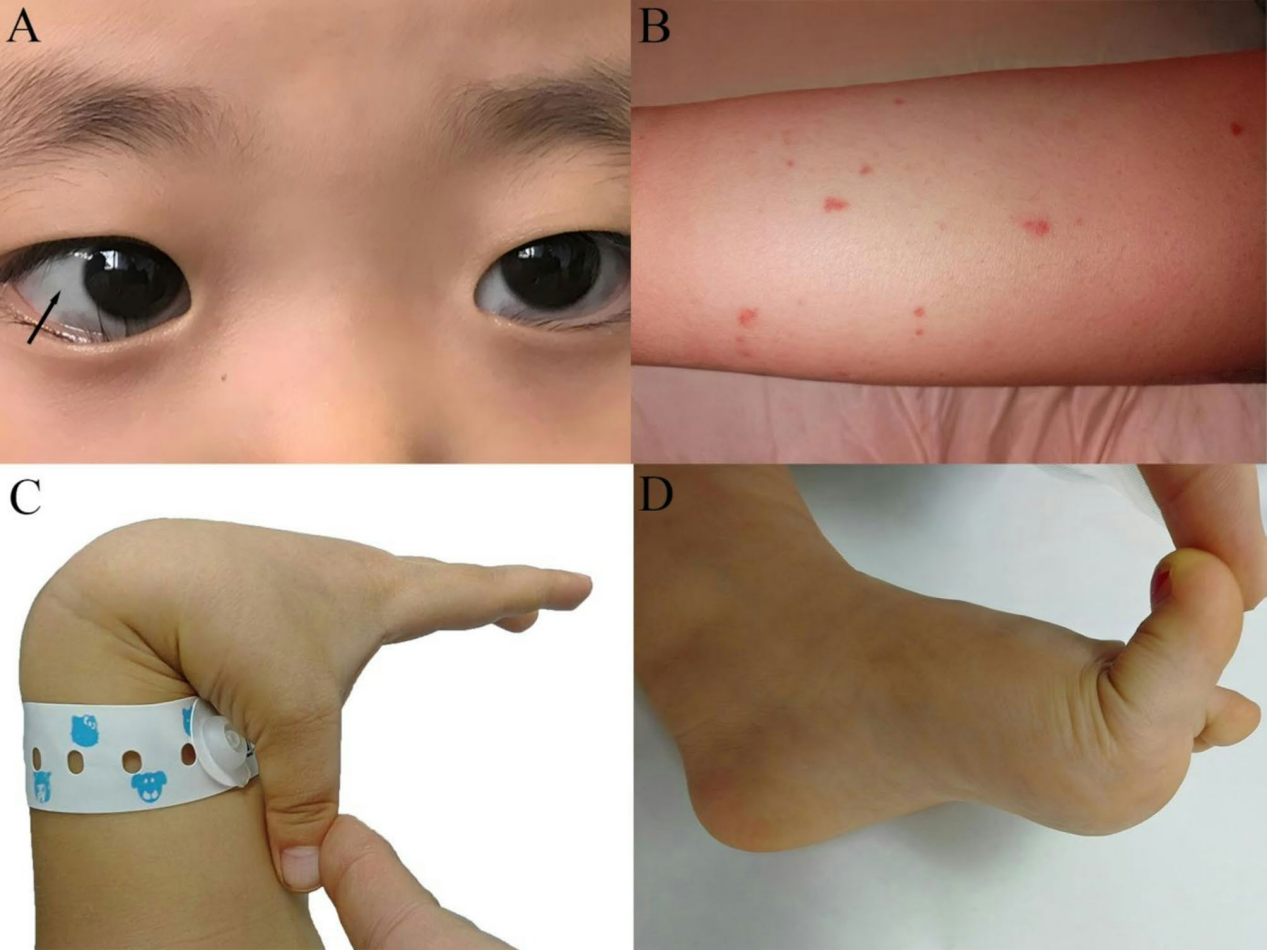
Clinical manifestations of patient 2 and patient 3, including: (**A**) blue sclera and (**B**) ecchymosis of calf in patient 2, (**C**), (**D**) hyperextension of multiple joints in patient 3

The clinical manifestations of patient 2 included blue sclera and joint hypermobility, consistent with EDS (Fig. 4A and B). The dermatopathological findings of patient 2 were also consistent with those of vEDS, showing collagen changes and focal elastic fiber breaks in the skin histopathology. This corroborates the idea that genetic mutations result in alterations in the size and distribution of major collagen fibrils and elastic fiber abnormalities [20]. In patient 2, the presence of numerous hemosiderin-laden macrophage in the bronchoalveolar lavage fluid confirmed the diagnosis of DAH. Patient 2 showed two heterozygous variants in the *COL3A1* gene, both of which originated from her mother. Although her mother did not experience alveolar hemorrhage, she experienced joint laxity, visible blood vessels in her chest, and epistaxis. Hemorrhagic events have been previously reported in mutation c.1804 C > A (p. Pro602Thr) [21], but not in another mutation c.1550 C > T (p. Pro517Leu). Both mutations resulted in the substitution of proline (Pro). Replacing Pro with other amino acids in type III collagen can significantly affect the structure and function of the protein (Additional File 2: Supplementary Fig. 1A). Due to its unique cyclic configuration, Pro is crucial for maintaining the stability of the triple helical structure. Consequently, Pro substitution can lead to loosening of the helix, reducing its thermal stability and mechanical strength. This structural instability can impair the functional performance of type III collagen in tissues, such as the skin and blood vessels, leading to abnormal accumulation or degradation of collagen and impacting tissue health. Type III collagen is a crucial component of the extracellular matrix, and maintaining the integrity and homeostasis of the extracellular matrix is essential for normal tissue development [22]. Chiarelli et al. [23] conducted a transcriptome analysis of skin fibroblasts from patients with vEDS caused by *COL3A1*. The analysis revealed significant changes in the expression levels of multiple genes involved in cell redox and endoplasmic reticulum homeostasis maintenance, collagen folding and extracellular matrix organization, proteasome complex formation, and cell cycle regulation. These results support that type III collagen could affect extracellular matrix integrity and homeostasis. Type III collagen interacts with type I and type II collagen in fiber formation [24]. In mice, type III collagen also plays a pivotal role in the generation of collagen fibers in dermal and arterial tissues [25]. Therefore, we suggest that at least one of the two mutations was associated with severe clinical presentation, including patchy limb hemorrhaging in early infancy and persistent DAH (7 years) in patient 2. Furthermore, both mutations may have played a synergistic role, resulting in severe and persistent DAH.

Patient 3 exhibited multiple hypermobile joints (Fig. 4C and D) and mutation c.3133G > A (p. Ala1045Thr) in the *COL3A1* gene originated from her father. He had a history of significant superficial upper extremity veins and interphalangeal and wrist joint hyperextension. Although the significance of this mutation is uncertain, two medical teams from Japan and China have reported vascular events caused by this mutation [26, 27]. This mutation is located in the tail of the triple helix region (Additional File 2: Supplementary Fig. 1B), which is adjacent to the C-terminal prepeptide, and hydroxyl introduction interferes with the prepeptide folding. Interchain disulfide bond (Cys1293-Cys1397) formation may be blocked, and trimer secretion efficiency may be reduced.

The mutation c.2573 C > G (p. Ala858Gly) in patient 4 originated from his mother, who had blue sclera, skin hyperextensibility, and joint hypermobility. This mutation involves a glycine residue, and the protein change was predicted to be harmful (Additional File 2: Supplementary Fig. 1C). Increasing the number of glycine pairs in type I collagen can result in protein misfolding and aggregation, which may increase flexibility and reduce tensile strength, thereby affecting tissue functional performance. Immunofluorescence findings revealed a lower amount of type I collagen compared to the control samples, and pulmonary pathology showed focal disruption of the local vessel walls, all indicating the pathogenicity of this mutation. The pulmonary pathology of patient 5 was consistent with that of EDS. The mutation c.3979G > A (p. Gly1327Ser) of the *COL1A1* gene originated from her father, who had visible skin vessels and mild joint hyperextensibility. Although this mutation has not been previously reported, the EDS Variant Database has indicated that the most common mutation is a majority of missense variants involving a glycine residue. Mutations of a non-Gly site to Gly and a Gly site to non-Gly result in structural abnormalities in type I collagen (Additional File 2: Supplementary Fig. 2A). Dimori et al. [28] previously indicated that type I collagen alterations have positive correlations between lung morphology and function and the severity of extracellular matrix deficiency in mice. The *COL1A2* c.3313G > A (p. Gly1105Ser) mutation in patient 6 originated from her father, who also had joint hypermobility. Junkiert-Czarnecka et al. [29] previously reported this missense mutation involving a glycine residue in a 14-year-old boy with skin hyperextensibility, generalized joint hypermobility, easy bruising, and a gothic palate. This missense mutation in Patient 6 is located at the end of the triple helix region (Additional File 2: Supplementary Fig. 2B), followed by the C-terminal sequence of the protein, which undergoes enzymatic cleavage during protein maturation. This mutation may cause shortening of the alpha helix around the C-terminal cleavage area, resulting in some of the helical regions becoming more flexible in the region of the cleavage site. This increased flexibility may make it more challenging for the enzyme to recognize the cleavage site.

The *TNXB* gene encodes tenascin XB, which regulates collagen deposition by dermal fibroblasts and plays a causative role in EDS [30]. Both patient 7 and his mother showed joint hypermobility, and the c.211G > T mutation originated from the mother. A study by Elahi et al. [31] reported the *TNXB* mutation c.211G > T in a patient with joint hypermobility. The dermatopathological and IHC findings of patient 7 were consistent with those of patients with EDS, and the lung pathological findings were consistent with the desmoid-like fibrous tissue reported by Varone et al. [32] Collectively, this mutation was considered likely pathogenic.

In addition to the clinical manifestations, the pulmonary pathology findings of patient 8 were consistent with those of vEDS. In particular, lung pathology showed uneven thickening of the small interstitial blood vessel walls, indicating structural abnormalities of the vasculature. The c.4691 C > A (p. Ser1564Tyr) mutation affects the C-terminal structure of the collagen triple helix trimer, which is essential for the proper assembly and maturation of collagen fibrils (Additional File 2: Supplementary Fig. 3). Type IV collagen is the primary constituent of the basement membranes of many tissues, including the vascular endothelium. Although this mutation and pulmonary hemorrhage have not been previously reported, the *COL4A2* gene has been associated with vascular events, such as cerebral hemorrhage [33–35]. Therefore, we hypothesized that *COL4A2* might be a potential gene associated with vEDS. Further basic experimental evidence is still necessary for confirming this.

The primary treatment for EDS is the restriction of activity. Some studies have speculated that administering ascorbic acid (vitamin C) may be beneficial as a cofactor in forming collagen fiber cross-links, thereby ameliorating the patient’s tendency to bruise [36]. Diuretics, beta-adrenergic receptor blockers, angiotensin receptor blockers, and other antihypertensive agents have also been proposed as treatments of vEDS [37]. In our study, glucocorticoids were administered to all patients in the acute phase until definitive diagnosis, and in the non-acute phase, hormonal therapy was less effective than immune-mediated pulmonary hemorrhage. Pulmonary hemorrhage improved significantly in patients 1 and 6 following activity restriction. Patient 4 was effectively treated for BAE. Vitamin C and E supplementation may have a therapeutic effect on bleeding caused by EDS. Patient 2’s persistent pulmonary hemorrhage was alleviated by strict activity restriction and vitamin C and E supplementation.

This study had some limitations, including its single-center retrospective design, the small number of participants, and the fact that IHC was not performed on all enrolled patients. Further multicenter studies are required to address these limitations.

## Conclusions

In the present study, we reported the relationship between non-vascular EDS and pulmonary hemorrhage and identified two novel mutations associated with vEDS. We added pathogenic evidences of two mutations in *COL3A1* gene and hypothesized *COL4A2* might be a potential gene for vEDS. These findings would enhance clinical recognition of EDS, particularly non-vascular EDS, and provide a sound basis to recommend that children with pulmonary hemorrhage be routinely examined for joint and skin hyperextension.

## Electronic supplementary material

Below is the link to the electronic supplementary material.

**Supplementary Material 1: Additional file 1: Supplementary table 1, Supplementary table 2**

**Supplementary Material 2: Additional file 2: Supplementary fig. 1, Supplementary fig. 2, Supplementary fig. 3**

## Data Availability

The datasets used and analyzed during the current study are available from the corresponding author upon reasonable request.

## Abbreviations

ACMG: American college of medical genetics and genomics
BAE: Bronchial artery embolization
CT: Computed tomography
DAH: Diffuse alveolar hemorrhage
EDS: Ehlers-Danlos syndrome
GGO: Ground glass opacities
IHC: Immunohistochemistry
vEDS: Vascular EDS
