# Supplementary material for: Pediatric pulmonary hemorrhage observed in non-vascular and vascular Ehlers–Danlos syndrome
Source: Orphanet J Rare Dis. 2025 Jul 1;20:329. doi: 10.1186/s13023-025-03858-2 (PMC12211803; doi:10.1186/s13023-025-03858-2)
Supplement: Supplementary file 1 — Supplementary Material 1: Additional file 1: Supplementary table 1, Supplementary table 2 [file 13023_2025_3858_MOESM1_ESM.docx]

**Pediatric Pulmonary Hemorrhage Observed in Non-Vascular and Vascular Ehlers–Danlos syndrome**

Supplementary Table 1: Characterization of laboratory tests of individual patients for differential diagnosis.

| 8 | 7 | 6 | 5 | 4 | 3 | 2 | 1 | Patient.No |
| --- | --- | --- | --- | --- | --- | --- | --- | --- |
| 42 | 106 | 40 | 96 | 79 | 35 | 36 | 127 | Minimum values of hemoglobin(g/L) |
| N | N | N | N | 21 | N | N | N | CRP(mg/L) |
| N | N | N | N | N | N | N | N | ANCA |
| ANA 1:80 | N | N | ANA 1:80 | N | N | N | N | Other autoantibodies |
| N | N | N | N | mildly elevated | N | mildly elevated | mildly elevated | Early Kidney Injury Markers |
| IgG, IgM, IgE increased; complement C3, C4, NK cells reduced | IgE increased | IgM, T cell increased; complement, NK cells reduced | N | IgE increased | complement C3 reduced | complement C3, C4 reduced | complement C4 increased | Immune Function Assessment |
| milk grade 3, wheat grade 1 | N | milk grade 2 | N | Alternaria Nees grade 3 | milk grade 1 | N | N | Allergens |
| N | N | N | N | N | N | N | N | Genetic Metabolic Disease Related Tests |

N=Negative, CRP=C-reactive protein, ANCA=Anti-neutrophil cytoplasmic antibodies, ANA=antinuclear antibodies.

Supplementary Table 2: Details of each patient subjected examinations and experiments.

| Patient No. | Dermato  pathology | Lung pathology | Immune  histochemical staining | Immunofluorescence analysis of COL1A1 expression in human skin fibroblasts | Genetic sequencing and evaluation of variants |
| --- | --- | --- | --- | --- | --- |
| 1 | √ |  | √ |  | √ |
| 2 | √ |  | √ |  | √ |
| 3 | √ |  |  |  | √ |
| 4 |  | √ |  | √ | √ |
| 5 |  | √ |  |  | √ |
| 6 |  |  |  |  | √ |
| 7 | √ | √ | √ |  | √ |
| 8 |  | √ |  |  | √ |
