# Supplementary material for: Pediatric pulmonary hemorrhage observed in non-vascular and vascular Ehlers–Danlos syndrome
Source: Orphanet J Rare Dis. 2025 Jul 1;20:329. doi: 10.1186/s13023-025-03858-2 (PMC12211803; doi:10.1186/s13023-025-03858-2)
Supplement: Supplementary file 2 — Supplementary Material 2: Additional file 2: Supplementary fig. 1, Supplementary fig. 2, Supplementary fig. 3 [file 13023_2025_3858_MOESM2_ESM.docx]

**Pediatric Pulmonary Hemorrhage Observed in Non-Vascular and Vascular Ehlers–Danlos syndrome**


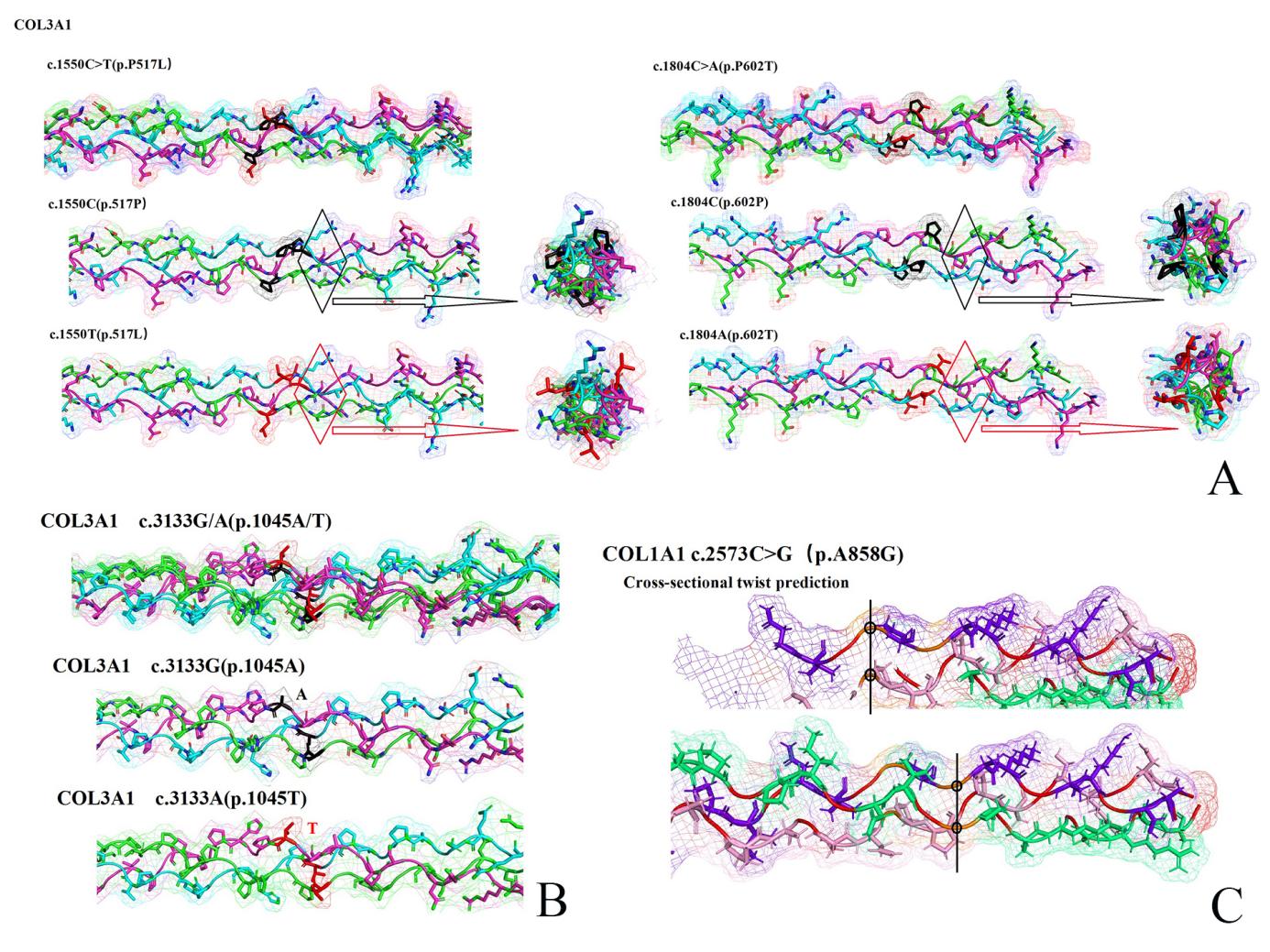


Supplementary Figure 1: Predicted proteins in patients 2, 3 and 4, including (A) patient 2 showed two heterozygous variants in the COL3A1 gene, involving the replacement of proline (Pro) with other amino acids, reducing its thermal stability and mechanical strength. (B) the mutation c. 3133G>A is located in the three-stranded helix region, and heterozygous mutations increase collagen bending by unbalancing the side chains. (C) the mutation c. 2573C>G increased glycine pairs (GG pairs), resulted in protein misfolding and aggregation, which may result in increased flexibility and reduced tensile strength.


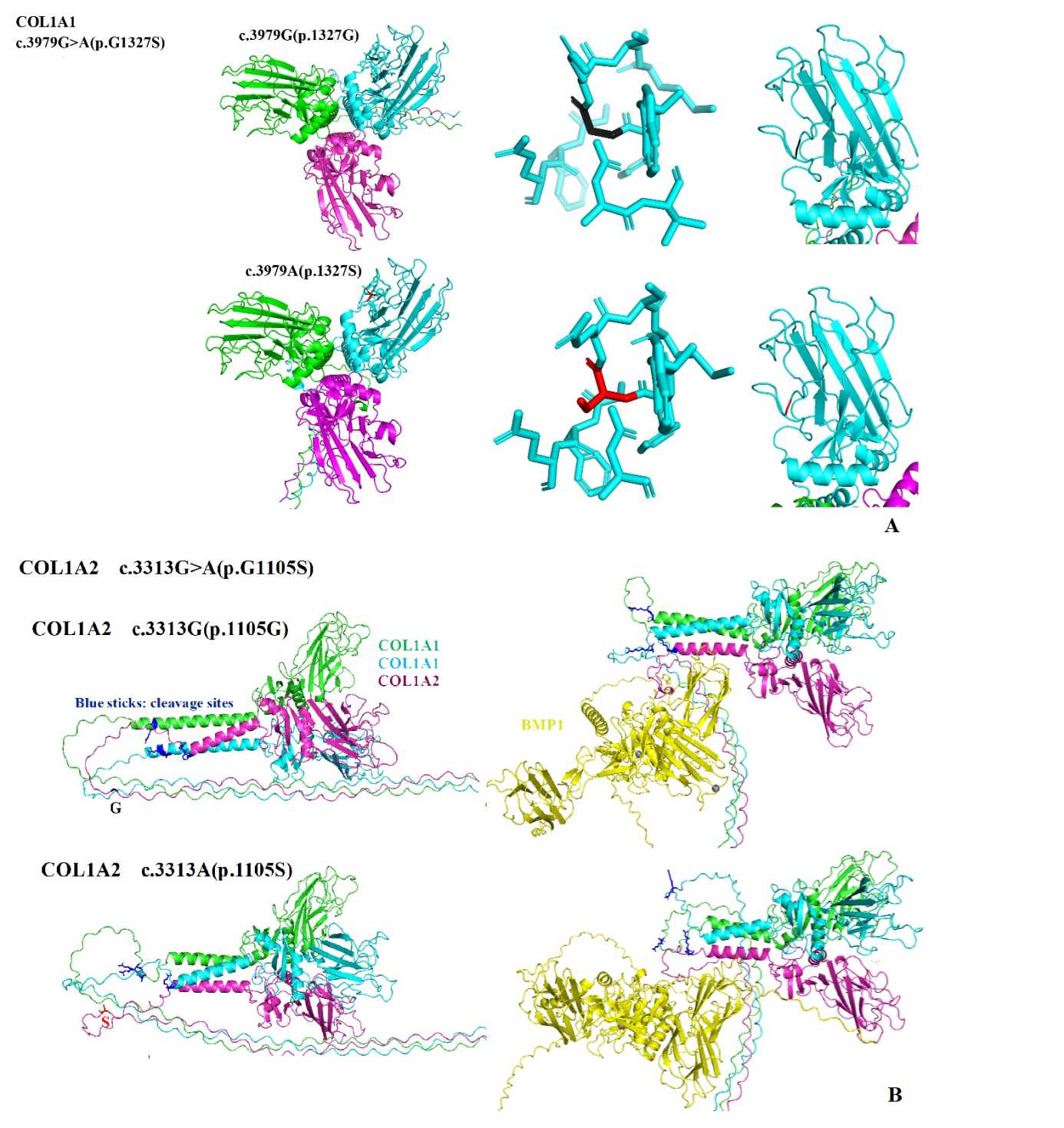


Supplementary Figure 2: Predicted proteins in patients 5 and 6, both the mutation c. 3979G>A of COL1A1 gene and c. 3313G>A of COL1A2 gene involving a glycine residue, a Gly site to non-Gly result in structural abnormalities in type I collagen. (A) The mutation c. 3979G>A is located in the Fibrillar collagen NC1 structural domain of the C-terminal prepeptide region and interferes with prepeptide folding and interchain disulfide bond formation. This may result in defective trimer assembly and decreased secretion. (B) The mutation c. 3313G>A may have caused a shortening of the alpha helix around the C-terminal cleavage area, resulting in some of the helical region becoming more flexible in the region of the cleavage site.


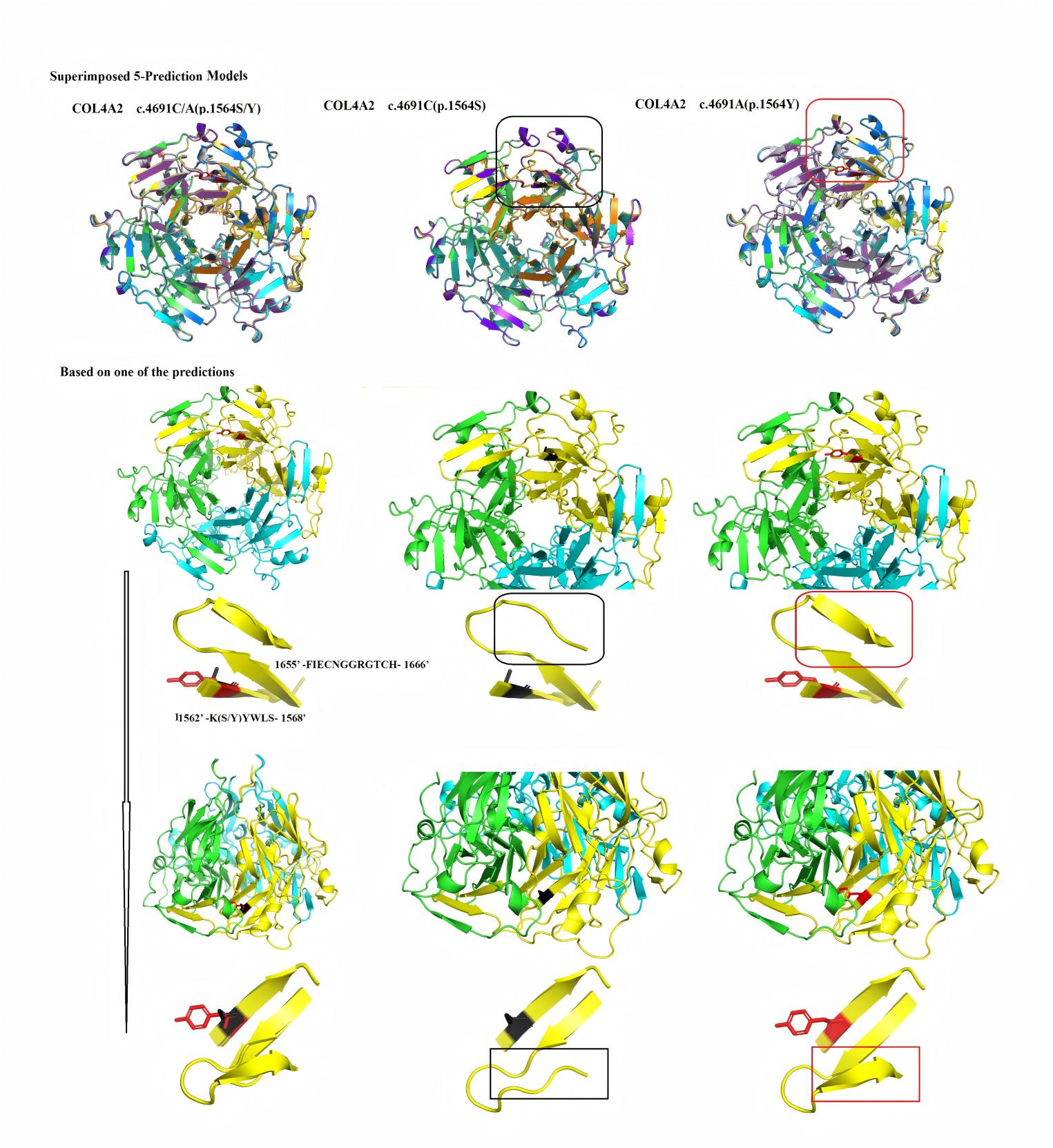


Supplementary Figure 3: Predicted proteins in patient 8. This mutation c. 4691C>A introduces bulky aromatic side chains that may interfere with the local α-helix or β-sheet conformation and reduce prepeptide stability. The hydroxyl (-OH) group of tyrosine may form abnormal intramolecular hydrogen bonds and disrupt the original hydrogen bonding network. If the mutation leads to a change in the conformation of the prepeptide, the pairing of COL4A1 and COL4A2 may be hindered, and the misfolded collagen may be retained in the endoplasmic reticulum, triggering the unfolded protein response and an imbalance of the proportion of type IV collagen in the extracellular matrix.
